# Cholinergic neurodegeneration and cholesterol metabolism dysregulation by constitutive p75^NTR^ signaling in the p75^exonIII^-KO mice

**DOI:** 10.3389/fnmol.2023.1237458

**Published:** 2023-10-13

**Authors:** Raquel Comaposada-Baró, Andrea Benito-Martínez, Juan Julian Escribano-Saiz, María Luisa Franco, Lorenzo Ceccarelli, Isabel Calatayud-Baselga, Helena Mira, Marçal Vilar

**Affiliations:** ^1^Molecular Basis of Neurodegeneration Unit of the Instituto de Biomedicina de Valencia CSIC, Valencia, Spain; ^2^Stem Cells and Aging Units of the Instituto de Biomedicina de Valencia CSIC, Valencia, Spain

**Keywords:** NGF (nerve growth factor), p75 neurotrophin receptor, TrkA (tropomyosin receptor kinase), cholinergic neurodegeneration, cholesterol, aging

## Abstract

Degeneration of basal forebrain cholinergic neurons (BFCNs) is a hallmark of Alzheimer’s disease (AD). However, few mouse models of AD recapitulate the neurodegeneration of the cholinergic system. The p75 neurotrophin receptor, p75^NTR^, has been associated with the degeneration of BFCNs in AD. The senescence-accelerated mouse prone number 8 (SAMP8) is a well-accepted model of accelerated and pathological aging. To gain a better understanding of the role of p75^NTR^ in the basal forebrain during aging, we generated a new mouse line, the SAMP8-p75^exonIII−/−^. Deletion of p75^NTR^ in the SAMP8 background induces an increase in the number of BFCNs at birth, followed by a rapid decline during aging compared to the C57/BL6 background. This decrease in the number of BFCNs correlates with a worsening in the Y-maze memory test at 6 months in the SAMP8-p75^exonIII−/−^. We found that SAMP8-p75^exonIII−/−^ and C57/BL6-p75^exonIII−/−^ mice expressed constitutively a short isoform of p75^NTR^ that correlates with an upregulation of the protein levels of SREBP2 and its targets, HMGCR and LDLR, in the BF of both SAMP8-p75^exonIII−/−^ and C57/BL6-p75^exonIII−/−^ mice. As the neurodegeneration of the cholinergic system and the dysregulation of cholesterol metabolism are implicated in AD, we postulate that the generated SAMP8-p75^exonIII−/−^ mouse strain might constitute a good model to study long-term cholinergic neurodegeneration in the CNS. In addition, our results support the role of p75^NTR^ signaling in cholesterol biosynthesis regulation.

## Introduction

Degeneration of basal forebrain cholinergic neurons (BFCNs) is a hallmark of Alzheimer’s disease (AD). BFCNs regulate a wide array of brain functions, including learning, memory, and attention (Niewiadomska et al., 2011; Ballinger et al., 2016; Allaway and Machold, 2017). BFCNs release acetylcholine (ACh) that plays an important role in memory function and it has been implicated in aging-related dementia. Loss of BFCNs is playing a significant role in cognitive dysfunction in AD (Mufson et al., 2003; Counts and Mufson, 2005; Marcello et al., 2012). Recent reports suggested that degeneration of cholinergic neurons precedes the cortical neurodegeneration observed in AD patients (Schliebs and Arendt, 2011; Fernández-Cabello et al., 2020). However, the degeneration mechanism of the cholinergic system is still unknown in part due to a lack of good animal models.

Neurotrophins regulate the survival of BFCNs through the activation of their receptors, p75^NTR^ and Trks (Boissiere et al., 1997; Boskovic et al., 2019). p75 neurotrophin receptor, p75^NTR^, is highly expressed in the BFCNs during all stages of their development. The normal function of p75^NTR^ within these neurons in the adult brain remains unclear (Coulson, 2006; Coulson et al., 2009; Qian et al., 2019). p75^NTR^ is a member of the tumor necrosis factor (TNF) receptor superfamily that regulates key biological processes in the nervous system (Ibáñez and Simi, 2012; Bothwell, 2014; Meeker and Williams, 2014) and plays several functions during the development and in the adult nervous system (Kraemer et al., 2014b). p75^NTR^ is best known for its role in programmed neuronal death during embryonic development or in response to injury (Ibáñez and Simi, 2012). However, it also regulates axonal growth and synaptic plasticity, as well as cell proliferation, migration, and survival (Kraemer et al., 2014b). These functions can be elicited by the association of p75^NTR^ with different ligands and co-receptors and the activation of various signaling pathways (Roux and Barker, 2002; Vilar, 2017). The role of p75^NTR^ in the survival of BFCNs has been studied in several models. In general, p75^NTR^ *knock-out* mice models showed that the number of choline acetyltransferase (ChAT)-positive neurons in the BF is increased at birth (Yeo et al., 1997; Van der Zee and Hagg, 1998; Ward and Hagg, 1999; Barrett et al., 2010; Boskovic et al., 2014), suggesting that during embryonic development p75^NTR^ might cause apoptosis in these neurons.

The senescence-accelerated mouse (SAM) strains are mouse models used for investigating the biochemical and physiological basis of pathological aging (Chiba et al., 2009; Akiguchi et al., 2017). The SAM models were established through phenotypic selection from a common genetic pool of AKR/J mice (Chiba et al., 2009; Liu et al., 2020). Among its prone sub-strains, the SAMP8 (SAM Prone 8) shows accelerated aging and features typical of age-related cognitive impairments, like increased oxidative stress, memory impairment, an increase of phospho-tau and soluble Amyloid beta peptide, Aβ (Morley, 2002). At the same time, the SAM Resistant (SAMR) mouse models were generated as aging-resistant controls.

Here we describe the generation of a new mouse model, the SAMP8-p75^exonIII−/−^ mouse, which exhibits BFCN neurodegeneration, cognitive deficiencies and cholesterol biosynthesis genes upregulation.

## Results

### Phenotypic characterization of SAMP8-p75^exonIII−/−^ mice

**Figure 1:**
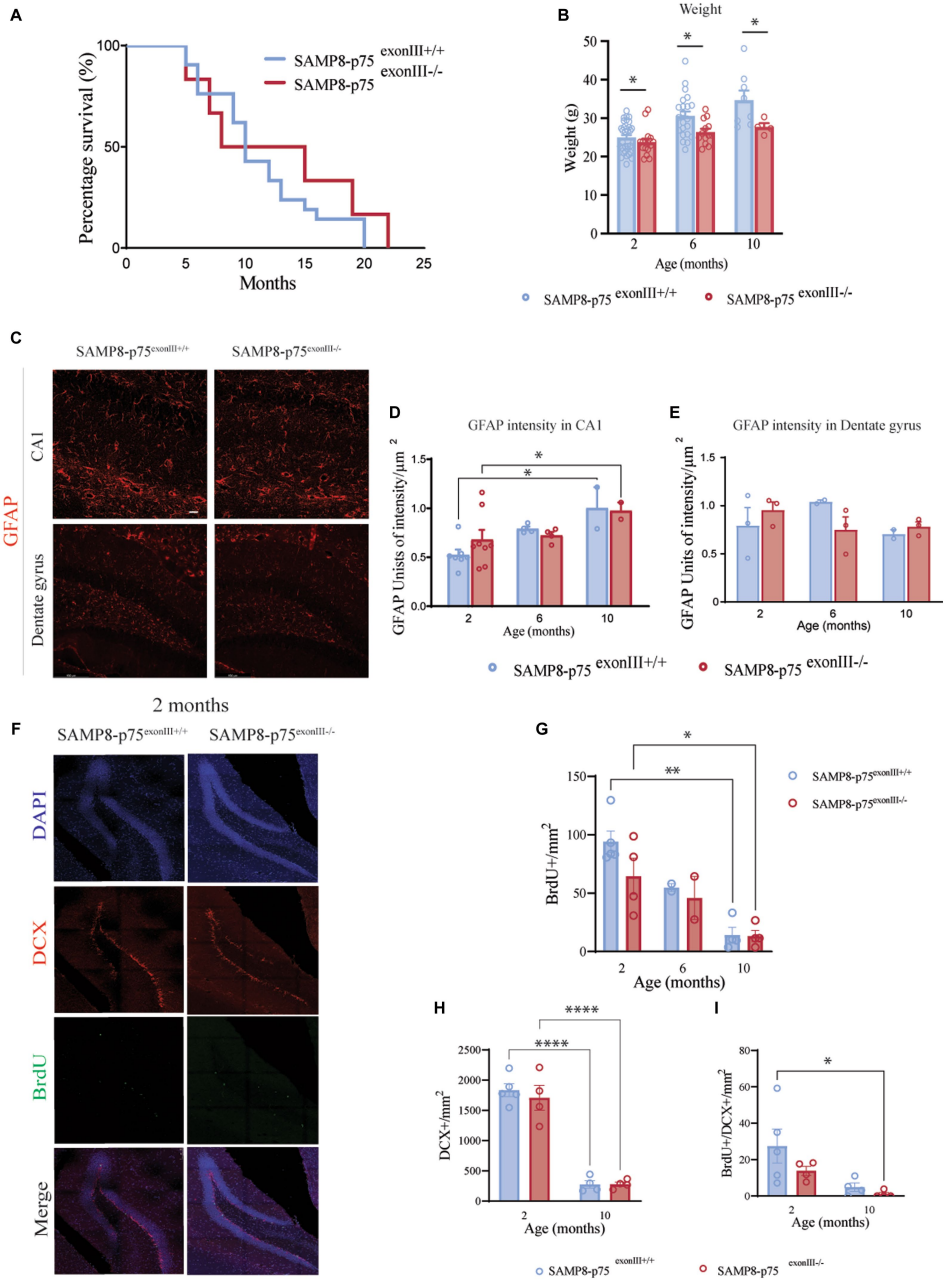
Characterization of SAMP8-p75^exonIII−/−^ mice. **(A)** Survival Kaplan plot showing no significant differences between SAMP8-p75^exonIII+/+^ (*N* = 21, mean half-life =10 months) and SAMP8-p75^exonIII−/−^ (*N* = 12, mean half-life = 11.5 months), Log-rank (Mantel-Cox) test, ns. **(B)** Bar plot showing the weight of the two mice genotypes at different ages. The bars represent the standard error of the mean, *N* = 8. Two-way ANOVA followed by Tukey’s posthoc analysis, ***p < 0.001. **(C)** Hippocampal astroglyosis. Representative images of the GFAP staining in the CA1 and dentate gyrus at 2 months. Scale bars, 25 μm. **(D)** Quantification of GFAP intensity in the CA1 region of the hippocampus at 2, 6, and 10 months. The bars represent the standard error of the mean, *N* > 3. Two-way ANOVA followed by Tukey’s posthoc analysis, **p < 0.05. **(E)** Quantification of GFAP intensity in the dentate gyrus region of the hippocampus at 2, 6, and 10 months. The bars represent the standard error of the mean, *N* > 3. Two-way ANOVA followed by Tukey’s posthoc analysis, **p < 0.01. **(F)** Hippocampal neurogenesis. Representative images of the staining of BrdU+/DCX+ cells in the SGZ of the dentate gyrus of SAMP8-p75exonIII+/+ and SAMP8-p75exonIII−/− mice; *N* = 4, four brain sections per animal. **(G)** Quantification of the number of BrdU+ cells in the SGZ of the dentate gyrus at 2, 6 and 10 months of age. **(H)** Quantification of the number of DCX+ cells in the SGZ of the dentate gyrus at 2 and 10 months of age. **(I)** Quantification of the number of BrdU+/DCX+ cells in the SGZ of the dentate gyrus at 2 and 10 months of age. Two-way ANOVA followed by Tukey’s posthoc analysis, *p < 0.05, **p < 0.01, ***p < 0.001, ****p < 0.0001.

SAMP8 was backcrossed during at least 12 generations with C57/BL6-p75^exonIII−/−^ (Lee et al., 1992). After 12 generations, heterozygotes SAMP8-p75^exonIII +/−^ mice were bred, and SAMP8-p75^exonIII+/+^ and SAMP8-p75 ^exonIII−/−^ littermates were selected for the aging studies. The same procedure was carried out to generate a SAMR1-p75 ^exonIII−/−^ mouse strain with the SAMR1 background, but we were unsuccessful for unknown reasons. SAMP8-p75 ^exonIII−/−^ mice showed a similar life survival (*p*-value, 0.51) respect to SAMP8-p75^exonIII+/+^ (Figure 1A).

SAMP8-p75 ^exonIII−/−^ mice showed a significant reduction in the total weight gain in a normal diet in comparison to the SAMP8-p75^exonIII+/+^ mice (Figure 1B). The SAMP8 strain has been reported to show an increase in astrogliosis in the hippocampus and in the cortex (Díaz-Moreno et al., 2013). The astrogliosis of SAMP8- p75 ^exonIII+/+^ and SAMP8-p75 ^exonIII−/−^ was quantified as the intensity of GFAP immunofluorescence in the CA1 region and in the dentate gyrus of the hippocampus (Figures 1C–E).An increase in the astrogliosis with age was found in the CA1 but no significant differences were observed between SAMP8-p75^exonIII+/+^ and SAMP8-p75 ^exonIII−/−^.

A previous work showed a reduction in adult hippocampal neurogenesis in the C57/BL6-p75^exonIII−/−^ strain (Catts et al., 2008) that was related to a reduction in the width of the hippocampal dentate gyrus granule cell layer, indicating a role of the p75^NTR^ in neurogenesis. We decided to characterize adult hippocampal neurogenesis in our newly generated model. In the case of the SAMP8 strain, there were no significant differences in the width of the granule cell layer (at 2 months SAMP8-p75^exonIII+/+^ 49.2 ± 2.5 μm, *N* = 5 vs. SAMP8-p75 ^exonIII−/−^ 46.2 ± 1.9 μm, *N* = 4; at 10 months SAMP8-p75^exonIII+/+^ 50.2 ± 9.2 μm *N* = 4 vs. SAMP8-p75 ^exonIII−/−^ 49.5 ± 0.3 μm *N* = 4), however, quantification of the number of BrdU^+^ cells showed that in the SAMP8-p75 ^exonIII−/−^ mice at the age of 2 months there was a slight decrease in the proliferative activity of the neural stem cell niche that did not reach statistical significance (Figures 1F–I). In addition, there was a reduction in the number of BrdU^+^/DCX^+^ newly born neurons in the subgranular zone (SGZ) of the dentate gyrus of the hippocampus during aging (from 2 to 10 months) and in the total number of immature DCX^+^ neurons irrespective of the mouse genotype (Figures 1H,I), supporting previous findings indicating that in aged SAMP8 mice, neurogenesis is impaired (Díaz-Moreno et al., 2013).

### Altered number of BFCNs in the SAMP8-p75^−/−^ mice

**Figure 2:**
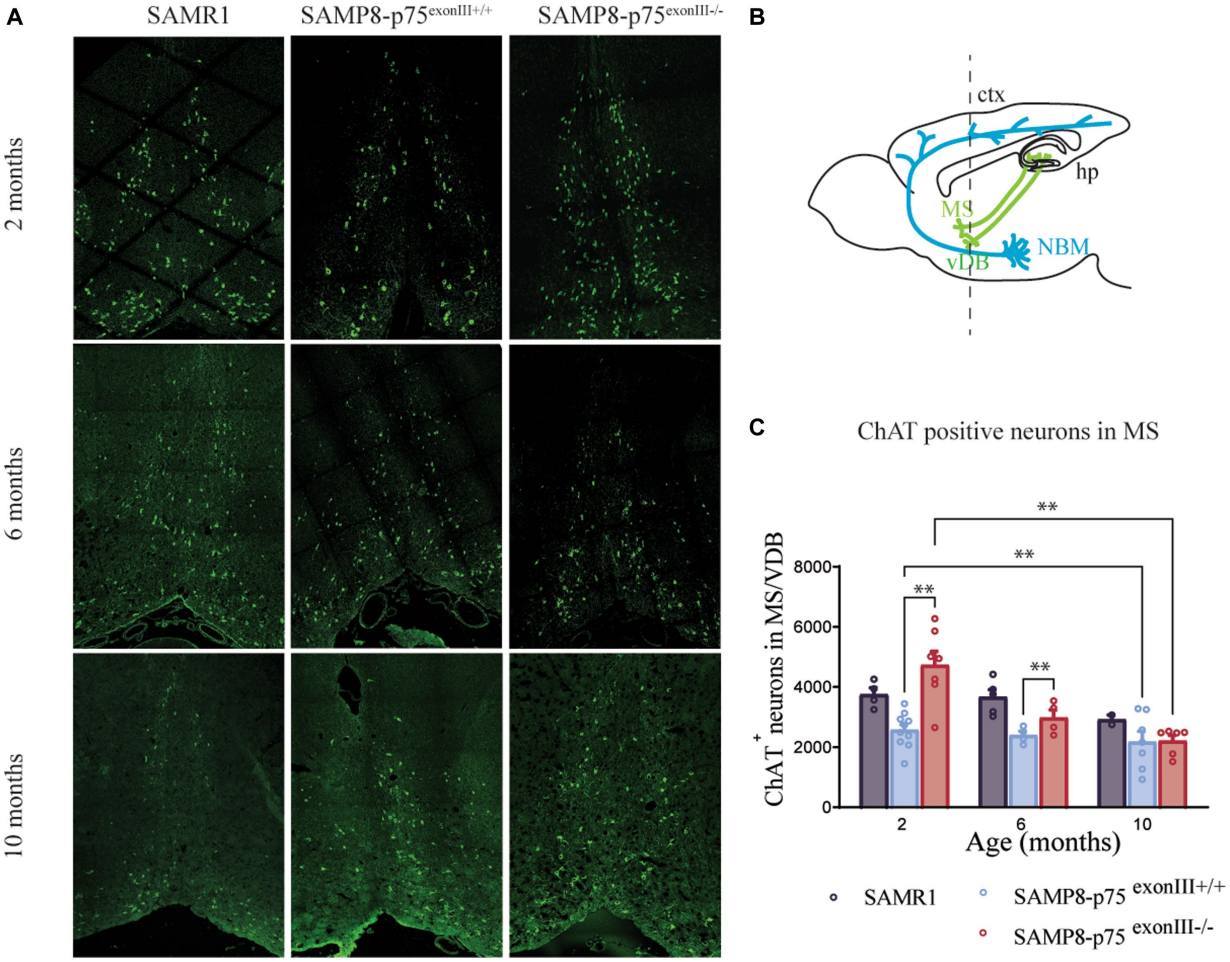
Number of medial septum and vertical diagonal band cholinergic neurons at different ages in SAMP8-p75^exonIII+/+^ and SAMP8-p75 ^exonIII−/−^ mice lines. **(A)** Representative image of medial septum cholinergic neurons stained with ChAT (green) at 2, 6 and 10 months of the different mice lines. **(B)** Mouse brain scheme showing the neuronal projections of the basal forebrain cholinergic neurons; ctx, cortex; hp., hippocamous; MS, Medial septum; NBM, nucleus basalis of Meynert; VDB, vertical diagonal band. **(C)** Quantification of ChAT-positive neurons in the MS/VDB at different ages. Mean ± _SEM, *N* < 4. Two-way ANOVA followed by Tukey’s posthoc test, *p < 0.05, ***p < 0.001, ****p < 0.0001.

As p75^NTR^ is highly expressed in the BFCNs, we focused on the study of this region (Figure 2). p75^NTR^ immunohistochemistry using an antibody against the intracellular domain was undertaken on BF sections. We quantified approximately 40% more ChAT^+^ neurons in SAMP8-p75^exonIII−/−^ than SAMP8-p75^exonIII+/+^ animals in the basal forebrain [medial septum (MS) and vertical diagonal band (VDB)] (Figures 2A–C). The number of the BFCNs in the SAMP8-p75 ^exonIII−/−^ is highest at 2 months of age, nevertheless, at 10 months of age, the two mouse genotypes showed the same number of BFCNs (Figure 2C), suggesting that the increased number of BFCNs in the SAMP8-p75^exonIII−/−^ degenerate or become ChAT-negative during this time interval.

**Figure 3:**
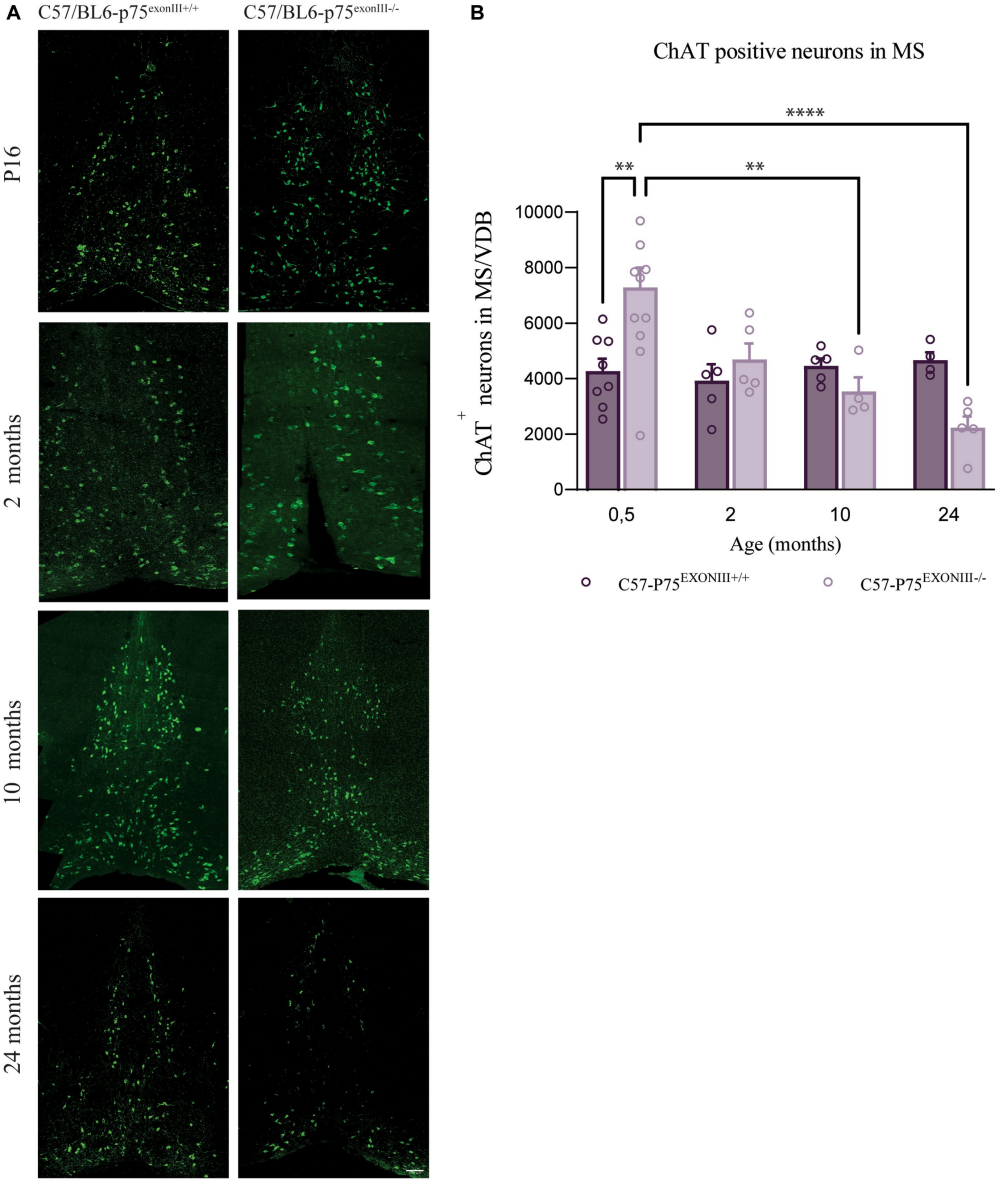
Number of medial septum and vertical diagonal band cholinergic neurons at different ages in C57/Bl6-p75^exonIII+/+^ and C57/Bl6-p75 ^exonIII−/−^ mice lines. **(A)** Representative image of medial septum cholinergic neurons stained with ChAT (green) at P16, 2, 10 and 24 months of the different mice lines. **(B)** Quantification of ChAT-positive neurons in the MS/VDB at different ages. Mean ± _SEM, *N* < 4. Two-way ANOVA followed by Tukey’s posthoc test, *p < 0.05, **p < 0.01.

To study if the accelerated decrease in the number of BFCNs in the SAMP8-p75^exonIII−/−^ is due to the mouse background strain, we compared the number of BFCNs in the C57/BL6-p75^exonIII−/−^ mouse strain (Figure 3). As the SAMP8 mice have accelerated aging, we quantified the number of BFCNs in C57/BL6-p75^exonIII+/+^ and C57/BL6-p75^exonIII−/−^ until geriatric ages (*ca.* 30 months old). We found that the number of BFCNs at birth is higher in the C57/BL6-p75^exonIII−/−^ similar to the SAMP8 background and to previous reports (Figures 3A,B) (Naumann et al., 2002), and the number of BFCNs slowly decreased and approached the same number than C57/BL6-p75^exonIII+/+^ at around 12 months old and reaching lower levels at 24 months of age (Figure 3B).

Altogether this indicates that the deletion of p75^NTR^ induces an increase in the number of BFCNs at birth, but in the long term, there is a decrease in the survival of BFCNs independent of the mouse strain (SAMP8 or C57/BL6). The loss of BFCNs in the SAMP8 background in comparison to the C57/BL6 background could be mediated by the specific characteristics of this accelerated aging mouse model strain.

### Deletion of p75^NTR^ in the SAMP8 mice has an impact on behavior

**Figure 4:**
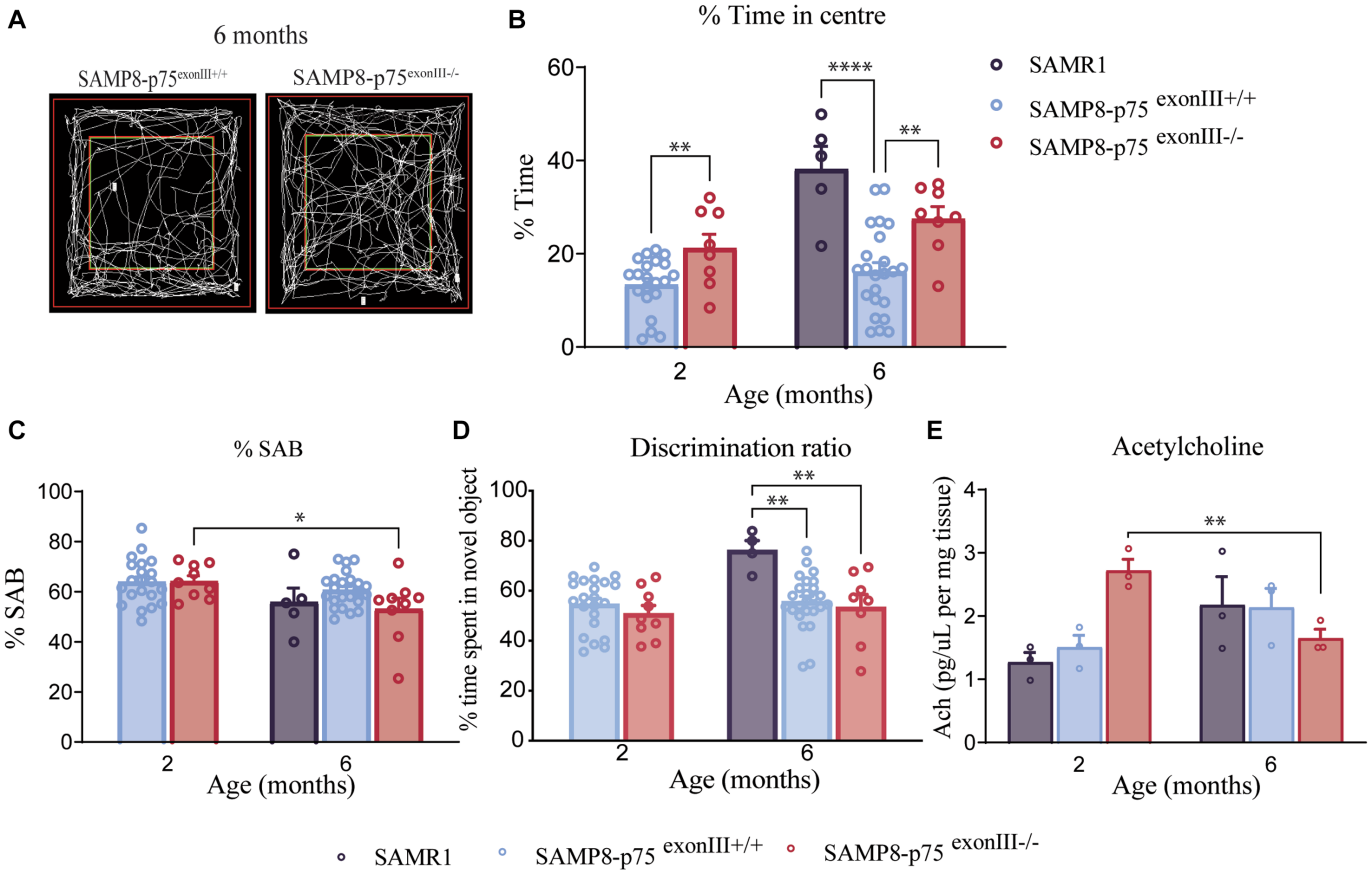
Behavior of SAMP8-p75 ^exonIII−/−^. SAMP8 anxiety decreases with p75^NTR^ deletion. **(A)** Representative images of the different open field measurements. **(B)** Open-field test. Percentage of the time spent in the center in the open field test. SAMP8 mice (blue and orange) are more anxiogenic than SAMR1 mice (green bar), however, SAMP8-p75 ^exonIII−/−^ partially decreases the anxiety levels. **(C)** Y-maze test. SAMP8-p75 ^exonIII−/−^ has a loss of spatial memory with age. Percentage of SAB (spontaneous alternation behavior) measured with the number of correct triplets divided by the total triplets. A difference from SAMP8-p75^exonIII+/+^ mice, SAMP8-p75^exonIII−/−^ loses episodic memory with age. **(D)** Novel-object recognition. The deletion of p75 in the SAMP8 mice has no effect on long-term memory. Percentage of time spent on the novel object. SAMP8 background showed a loss in long-term memory at 6 months compared to SAMR1 mice. SAMR1 6 months *N* = 5, SAMP8-p75^exonIII+/+^ 2 months *N* = 22, SAMP8-p75^exonIII−/−^ 2 months *N* = 8, SAMP8-p75^exonIII+/+^ 6 months *N* = 24, SAMP8-p75^exonIII−/−^ 6 months *N* = 8. Two-way ANOVA followed by Tukey’s post-hoc test, *p < 0.05, **p < 0.01, ***p < 0.001. **(E)** Levels of acetylcholine analyzed by HPLC and normalized per mg of tissue. *N* = 3. Two-way ANOVA followed by Tukey’s post-hoc test, **p < 0.01.

To determine whether p75^NTR^ deficiency in the SAMP8 mice affected anxiety and cognitive ability, a cohort of mice were subjected to the following behavioral tests: open-field (OF), Y-maze (YM) and novel object recognition (NOR). In order to assess the role of BFCNs, mice were tested at two different ages, 2 and 6 months. In the OF test, mice are tested for anxiety-related parameters measured as time spent in the center of the box. As animals display a natural aversion to brightly open areas (central zone) but the also have a dive to explore new environments (Kovacsics and Gould, 2010). A significant difference in the % of time spent in the center in the OF test was observed at both 2 and 6 months of age between SAMP8-p75^exonIII−/−^ and SAMP8-p75^exonIII+/+^ animals (Figures 4A,B), showing an increase of time the SAMP8-p75^exonIII−/−^ animals. The behavior of SAMP8-p75^exonIII−/−^ in the OF is similar to the control mice SAMR1, indicating that the deletion of p75 ^NTR^ in the SAMP8 rescues the behavior of the SAMP8-p75^exonIII+/+^ mice in this test. The SAMR1 has more BFCNs than the SAMP8 mice at this age and is more similar in number to the SAMP8-p75^exonIII−/−^ (Figure 2C), suggesting that the increased cholinergic innervation from the BFCNs may play a role in this phenotype. During the YM test, mice are positioned within the central point of a maze comprising three opaque arms, arranged in the configuration of the capital letter Y. Mice are allowed to freely explore their surroundings and due to their innate inclination to discover novel environments, the rodents exhibit a preference for exploring new arms of the maze. The primary objective of this test is to test their episodic memory, which is quantified by observing how often a mouse selects a previously unexplored arm of the maze (considered as the correct behavior). This parameter is referred to as SAB (spontaneous alternation behavior), a measure of the mouse’s tendency to alternate between arms without any external cues (Kraeuter et al., 2019). At 2 months of age, SAMP8-p75^exonIII+/+^ and SAMP8-p75^exonIII−/−^ mice were not behaving significantly differently (Figure 4C). However, at 6 months of age, the SAMP8-p75^exonIII−/−^ performed significantly worse than at 2 months of age, quantified with a lower % of correct alternations suggesting a worsening of these cognitive abilities. The decrease in the number of BFCNs from 2 to 6 months (around 50%) in SAMP8-p75^exonIII−/−^ may be responsible for this worsening in cognitive ability. This difference is not observed in the SAMP8-p75^exonIII+/+^ mice during aging from 2 to 6 months (Figure 4C), which did not display a reduction in the number of BFCNs during this time frame. In the NOR test, the preference for exploring new objects over familiar ones evaluates short and long memory. The test requires a training phase, where familiar objects are presented. This study specifically focused on evaluating long-term memory as the test phase is performed 24 h after the training phase. When tested in NOR (Figure 4D) there were no differences between SAMP8-p75^exonIII+/+^ and SAMP8-p75^exonIII−/−^ in the time exploring the novel object, but these two genotypes clearly performed worse than the control mice SAMR1 (Figure 4D). The decrease in the number of cholinergic neurons may impact on the release of the acetylcholine neurotransmitter. We measured the levels of acetylcholine in the different mice genotypes at the two ages of the study (Figure 4E). As shown, acetylcholine levels decrease in the SAMP8-p75^exonIII−/−^ from 2 to 6 months but not in the other genotypes (SAMR1 or SAMP8-p75^exonIII+/+^), suggesting that the decrease in the number of cholinergic neurons impact on the synthesis of acetylcholine and in the behavior.

Altogether the deletion of p75^NTR^ in the pathological strain SAMP8 induces differences in some of the studied tests, probably depending on the neuronal circuit involved.

### SAMP8-p75^exonIII−/−^ constitutively expresses a short isoform of p75^NTR^

**Figure 5:**
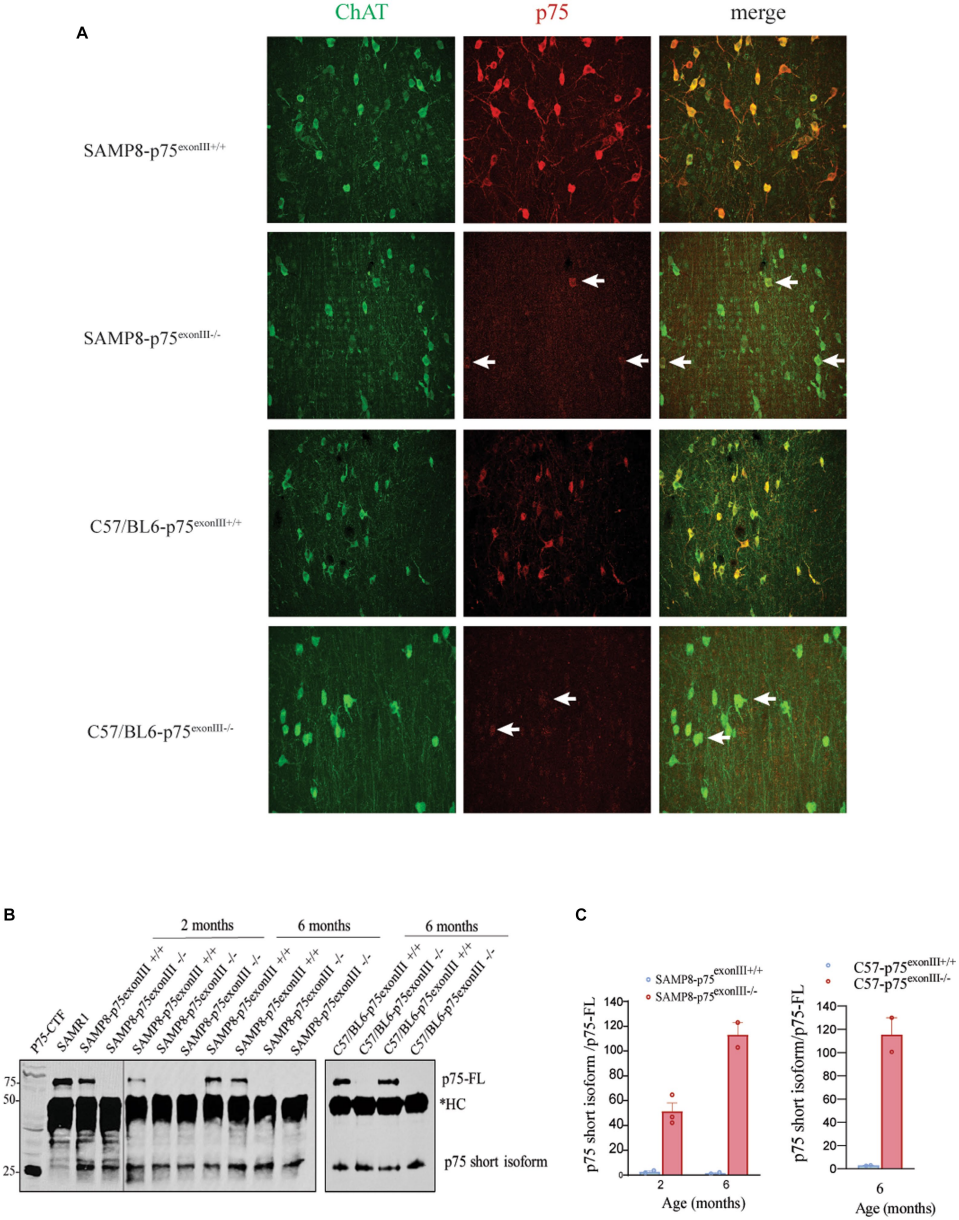
SAMP8-p75 ^exonIII−/−^ expresses a short isoform of p75^NTR^ in the cholinergic neurons. **(A)** Immunostaining of basal forebrain MS sections with a p75^NTR^ intracellular antibody showing a significant signal in the ChAT+ neurons from the SAMP8-p75^exonIII−/−^ mice. Arrows point to cholinergic neurons stained for ChAT and p75, *N* = 3. **(B)** Western blot of lysates immunoprecipitated with a p75 intracellular antibody in the SAMP8-p75 ^exonIII+/+^ and SAMP8-p75 ^exonIII−/−^ and in the C57/BL6-p75 ^exonIII+/+^ and C57/BL6-p75 ^exonIII−/−^ lysates, *HC, antibody heavy chain, *N* = 3. **(C)** Quantification of the ratio p75_short_isoform/p75-FL from western blots as showed in B in the SAMP8-p75 ^exonIII+/+^ and SAMP8-p75 ^exonIII−/−^ (left) and in the C57/BL6-p75 ^exonIII+/+^ and C57/BL6-p75 ^exonIII−/−^ (right) lysates.

The phenotype described in the SAMP8-p75^exonIII−/−^ suggests a deleterious effect of the deletion of p75^NTR^ on the survival of the BFCNs. Although these data suggest a pro-survival role of p75^NTR^, it has been described that depending on the mouse strain, the p75^exonIII−/−^ mice express a short isoform of p75^NTR^ that may play a negative impact on the neurons where it is expressed (von Schack et al., 2001). We analyzed this possibility in the SAMP8 background and found that in the SAMP8-p75^exonIII−/−^, there was a significant signal in the BFCNs when stained using a specific antibody against the intracellular domain (ICD) of p75^NTR^ (Figure 5A). To confirm this observation, we performed immunoprecipitation using a p75^NTR^ -ICD antibody and western blots analysis of basal forebrain tissue from 2- and 6-months old mice. The Figure 5B shows the presence of a short isoform of p75^NTR^ in the SAMP8-p75^exonIII−/−^. This short isoform migrates at a similar size as the p75^NTR^-C-terminal fragment, p75^NTR^-CTF, construct analyzed in the same blot (Figure 5B) and is reminiscent of a short isoform previously described in von Schack et al. (2001). Interestingly the expression of this short isoform is also observed in the C57/BL6-p75^exonIII−/−^ mice by immunostaining and co-immunoprecipitation (Figures 5A,B), indicating that the expression of the short isoform is due to the p75^exonIII^ genetic cassette construct and not dependent on the mouse strain. As p75^NTR^ undergoes receptor intramembrane proteoysis (RIP) in physiological conditions, the p75^NTR^-CTF is also observed in the wt mice (both SAMP8 and C57/BL6 strains) together with the p75^NTR^ full length (p75-FL) protein (Figure 5B). Quantification of the ratio p75-CTF/p75-FL (Figure 5C) indicates that there is a significant increase of this ratio in the SAMP8-p75^exonIII−/−^ and C57/BL6-p75^exonIII−/−^ mice that might play an aberrant signaling in these cells.

### Increase of the cholesterol biosynthesis genes in the basal forebrain of SAMP8-p75^exonIII−/−^

**Figure 6:**
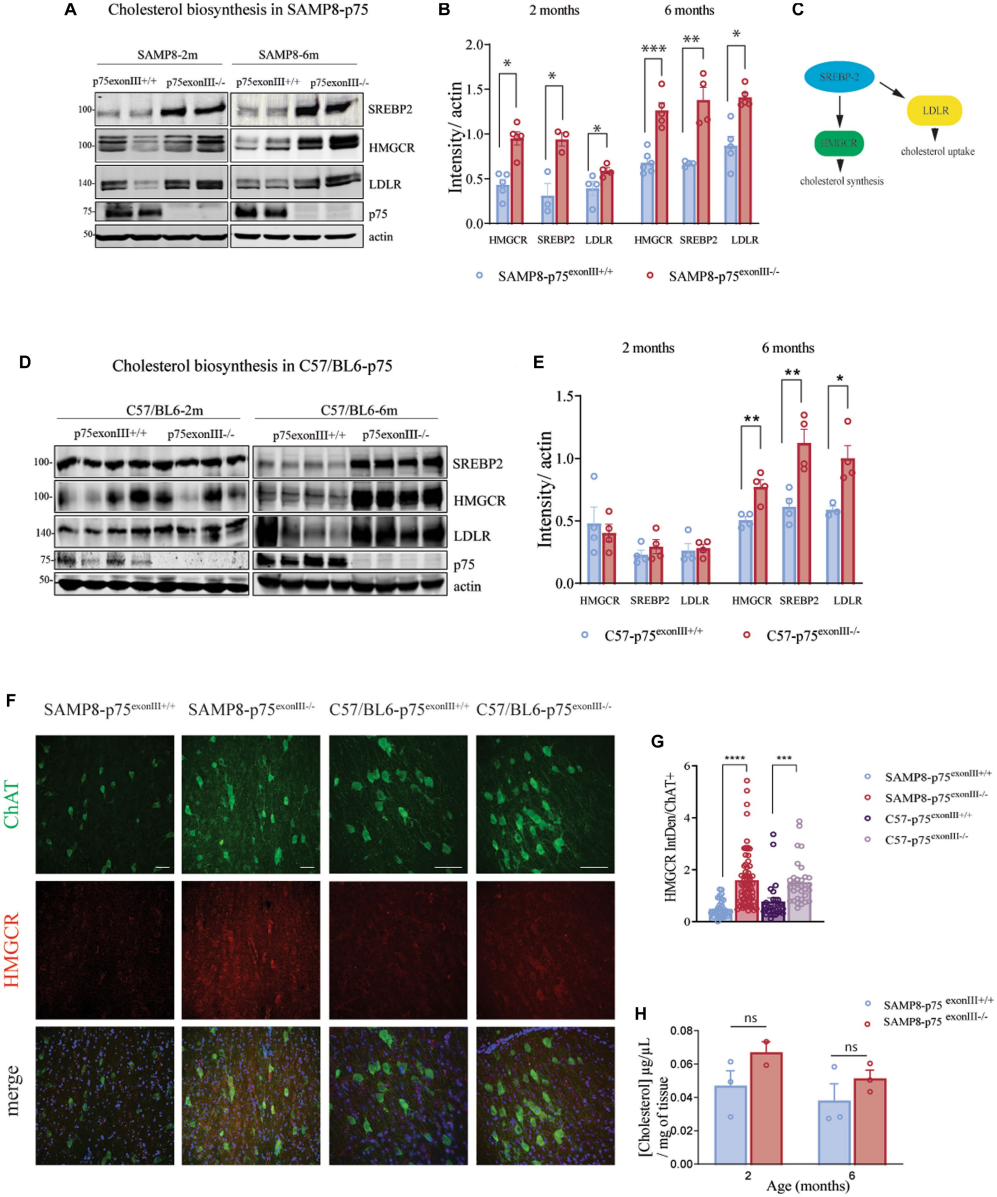
Upregulation of cholesterol biosynthesis genes in the p75^exonIII −/−^ mice strains. **(A)** Western blot analysis of basal forebrain extracts from 2 and 6 months old SAMP8-p75^exonIII+/+^ and SAMP8-p75^exonIII−/−^ mice showing expression of SREBP2, HMGRC, and LDLR (*N* = 4). **(B)** Quantification of the western blots showed in **(B)**. **(C)** Scheme showing the relationship between SREBP2, LDLR, and HMGRC. **(D)** Western blot analysis of basal forebrain extracts from 2 and 6 months old C57BL6-p75^exonIII+/+^ and C57BL6-p75^exonIII−/−^ mice showing expression of SREBP2, HMGRC and LDLR (*N* = 4). **(E)** Quantification of the western blots showed in **(D)**. **(F)** Immunofluorescence of basal forebrain slides showing the co-expression of ChAT and HMGRC in SAMP8-p75^exonIII−/−^ and 6 months old C57BL6-p75^exonIII−/−^ Scale bars, 50 μm. **(G)** Quantification of HMGCR levels in ChAT positive neurons. Each point indicates a ChAT positive cell. Statistical analysis was performed using a *t*-test, ***p < 0.001, ****p > 0.0001. **(H)** Cholesterol levels of basal forebrain extracts from SAMP8-p75^exonIII+/+^ and SAMP8-p75^exonIII−/−^ mice at 2 and 6 months old.

It has been described that p75^NTR^ regulates the metabolism of cholesterol in the nervous system (Yan et al., 2005; Korade et al., 2007; Follis et al., 2021) among other tissues (Pham et al., 2019). We quantified the levels of Sterol Regulatory Element-binding Protein-2, SREBP2, a transcription factor involved in the upregulation of key genes for cholesterol biosynthesis and cholesterol uptake (Figure 6A). As it is shown in the Figure 6B analysis by western blot of basal forebrain tissue showed a significant increase in the total expression of SREBP2 in the p75^exonIII−/−^ with respect to p75^exonIII+/+^ in both SAMP8 and C57/BL6 mouse strain. SREBP2 plays an important role in the homeostasis of cholesterol by regulating the 3-hydroxy-3-methylglutaryl-coenzyme A, HMGCR, a key enzyme in the synthesis of cholesterol and the low-density lipoprotein receptor, LDLR, that mediates the uptake of extracellular cholesterol. As shown in Figure 6B, the protein levels of HMGCR and LDLR increase significantly at 2 and 6 months in the in the SAMP8-p75^exonIII−/−^ versus SAMP8-p75^exonIII+/+^. In the case of the C57/BL6 mice, there is a significant increase at 6 months but not at 2 months. This difference could be related to the fact that the SAMP8 mice have an accelerated aging in comparison to the normal aging of the C57/Bl6. In any case at 6 months old, a similar increase of SREBP2, HMGCR and LDLR is observed indicating that is a general mechanism of the p75^exonIII−/−^ mice and not of the mouse background.

The cellular type responsible of HMGCR expression is important, as it is described that in the adult brain the synthesis of cholesterol takes place mainly in the astrocytes. Immunofluorescence instead showed an increase of the HMGRC staining in the neurons of the basal forebrain almost exclusively in the ChAT+ neurons of the BF of both SAMP8- p75^exonIII−/−^ and in the C57/BL6-p75^exonIII−/−^ mice (Figure 6F), supporting the western blot data. We then analyzed the total levels of cholesterol in basal forebrain extracts from SAMR1, SAMP8-p75^exonIII−/−^, and SAMP8-p75^exonIII+/+^ at 2 and 6 months (Figure 6H) and found an increase in the total levels of cholesterol, although not reaching statistical significant values, in the SAMP8-p75^exonIII−/−^ at 2 and a 6 months of age respect to SAMP8-p75^exonIII+/+^.

### The axis NGF/TrkA and p75^NTR^ regulates the expression of cholesterol biosynthesis genes

**Figure 7:**
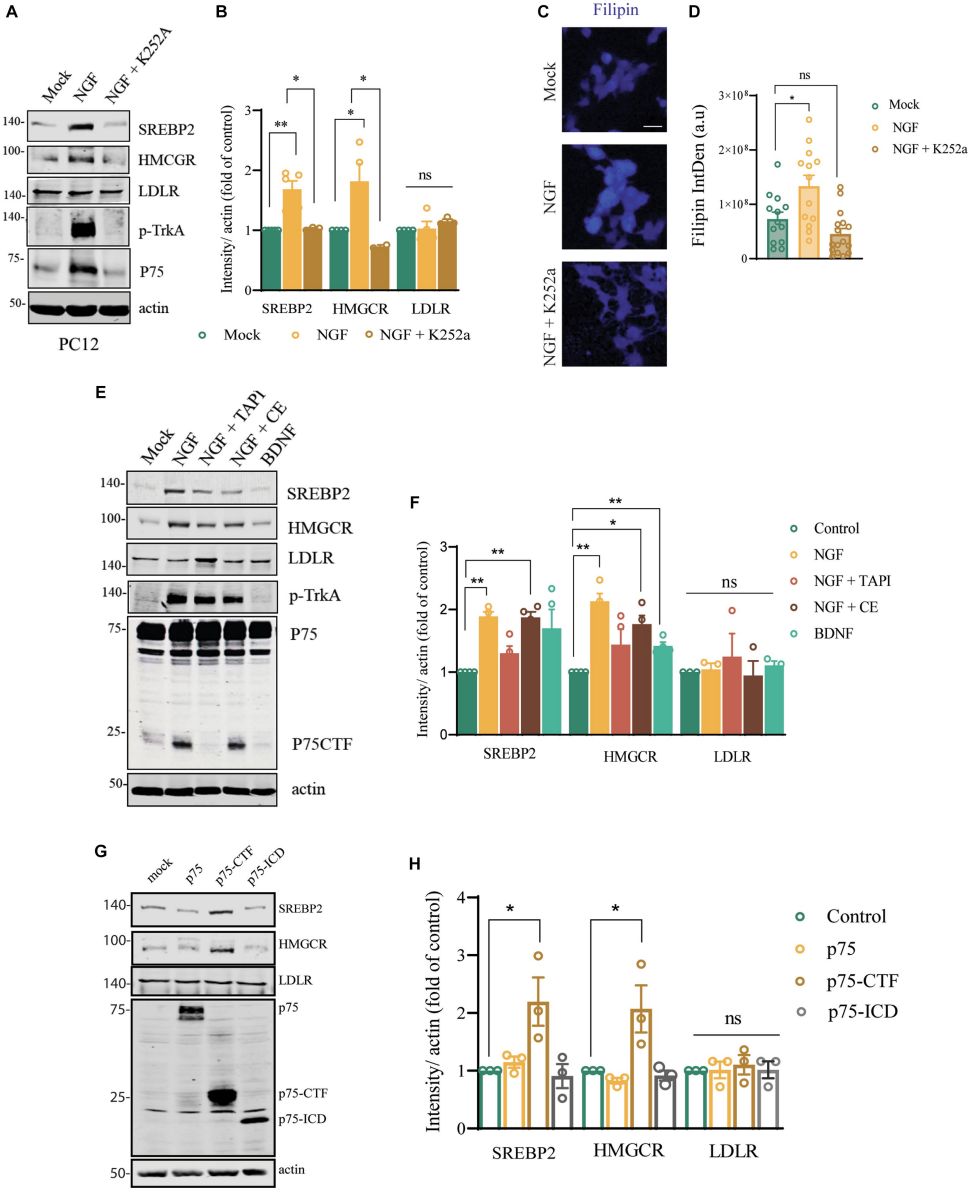
Cleavage of p75 mediated by TrkA/NGF signaling modulates cholesterol biosynthesis genes in PC12 cells. **(A)** Western blot analysis of PC12 lysates stimulated with NGF (50 ng/mL) for 24 h showing the expression of SREBP2, HMGRC, LDLR, p-TrkA and p75. **(B)** Quantification of the western blot showed in **(A)**. Mean ± _SEM, *N* < 4. For data presented as a fold increase, the One-sample *t* test and paired *t*-test was employed, *p < 0.05, **p < 0.01, ***p < 0.001. **(C)** Filipin staining of PC12 stimulated with buffer, NGF (50 ng/mL) or NGF + K252a for 24 h. **(D)** Filipin intensity quantification. Each point represents a single cell. One-way ANOVA followed by Tukey’s posthoc test, *p < 0.05. **(E)** Western blot analysis of PC12 lysates stimulated with NGF (50 ng/mL) and TAPI-1, Compound E or BDNF for 24 h showing the expression of SREBP2, HMGRC, LDLR, p-TrkA and p75. **(F)** Quantification of the western blot showed in **(D)**. Mean ± _SEM, *N* < 4. For data presented as a fold increase, the One-sample *t* test and paired *t*-test was employed *p < 0.05, **p < 0.01, ***p < 0.001. **(G)** Western blot analysis of PC12-TrkA/p75-DKO cells transfected with mock, p75 full length, p75-CTF and p75-ICD showing the expression of SREBP2, HMGRC, LDLR and p75. **(H)** Quantification of the western blot showed in **(G)**. Mean ± _SEM, *N* < 4. The One-sample *t* test and paired *t*-test was employed, *p < 0.05, **p < 0.01, ***p < 0.001.

So far, we have described a correlation between the presence of a short isoform of p75^NTR^ in the p75^exonIII^ mice (independent of the mice strain) and an increase in the cholesterol biosynthetic proteins in the BFCNs. To demonstrate that the neurotrophin signaling is able to activate these genes, we used a heterologous system like the PC12 cells that express endogenous levels of p75^NTR^ and TrkA, stimulated with NGF for 24–48 h (Figure 7). Stimulation of NGF induces an increase in the expression of SREBP2 and HMGRC in 24 h but not of LDLR (Figures 7A,B) and an increase in the cholesterol content, as shown by filipin staining (Figures 7C,D). NGF/TrkA stimulation also induces p75^NTR^ upregulation (Figure 7A). Interestingly NGF stimulation in the presence of a TrkA kinase activity inhibitor, K252a, prevents the activation of these genes (Figures 7A,B) and the increase of cholesterol content measured by filipin staining (Figure 7C). As TrkA activation by NGF induces the regulated intramembrane proteolysis (RIP) of p75^NTR^, we incubated the PC12 cells in the presence of an α-secretase inhibitor (TAPI-1) or a γ-secretase inhibitor (Compound E) (Figures 7E,F). Inhibition of p75^NTR^ shedding with TAPI-1 reduces the expression of HMGCR induced by NGF/TrkA, suggesting that shedding of p75^NTR^ is required for the upregulation of HMGCR. The presence of a γ-secretase inhibitor partially impairs the upregulation of HMGCR, indicating that the RIP of p75^NTR^ plays a role in HMGCR upregulation. Activation of p75^NTR^ independently of TrkA with BDNF (Figures 7E,F), is not able to upregulate the expression of HMGCR, supporting the data that TrkA activity is required. These experiments suggested that the activation of TrkA by NGF is required for the shedding of p75^NTR^, which is the main driver of the upregulation of the cholesterol biosynthesis genes. In order to demonstrate the direct role of p75^NTR^-CTF in this process we overexpressed p75^NTR^, p75^NTR^-CTF and p75-ICD in PC12TrkA/p75DKO cells (Testa et al., 2022) and found and increase in SREBP2 and HMGCR expression independently of NGF and TrkA (Figures 7G,H) in the cells overexpressing p75-CTF suggesting that accumulation of p75-CTF is a causative agent and pointing to the findings observed *in vivo* in the SAMP8-p75^exonIII−/−^ and C57/BL6-p75^exonIII−/−^ mice (Figure 6).

## Discussion

BFCNs are involved in several cognitive tasks (Ballinger et al., 2016). In humans, it has been described that a reduction of the cholinergic area is a general hallmark of Alzheimer’s disease patients (Mufson et al., 2008; Schliebs and Arendt, 2011; Kerbler et al., 2015; Fernández-Cabello et al., 2020). Here we found that the SAMP8-p75^exonIII−/−^ mouse is a good mouse model for studying cholinergic neurodegeneration. The role of p75^NTR^ in the BFCNs has been the focus of intense research since the initial observation that p75^NTR^ is highly expressed in these neurons (Ward and Hagg, 1999). Analysis of the p75^NTR^ knock-out mice showed an increase in the number of BFCNs at birth. This finding has been observed whatever deletion strategy was performed; in the initial p75^exonIII−/−^, p75^exonIV−/−^ deleted mice and in the more recent conditional mice with p75 deleted in the ChAT-expressing cells (Peterson et al., 1997; Yeo et al., 1997; Greferath et al., 2000; Naumann et al., 2002; Boskovic et al., 2014). These findings suggested that p75^NTR^ plays a critical role in the total number of BFCNs at birth. Although it has been proposed that this is indicative of a pro-apoptotic role of p75^NTR^ in this neuronal population during embryonic development, it has not been fully demonstrated with the use of an apoptotic-impaired conditional mice, for instance. The increase of BFCNs in the p75^NTR^ knock-out mice could also be the result of an increase in proliferation or due to a positive role of p75^NTR^ in the neuronal precursor’s mitotic exit. This could be similar to the role of p75^NTR^ in the mitotic exit found in the cerebellar granule cell progenitors (GCP) where in the absence of p75^NTR^, GCPs continue to proliferate beyond their normal period, resulting in a larger cerebellum that persists into adulthood (Zanin et al., 2016). Here we have observed that deletion of p75^NTR^ in the SAMP8 background, a pathological aging mouse model, also results in an increase in the number of BFCNs at birth as it was reported in other mice strains (Peterson et al., 1997; Yeo et al., 1997; Greferath et al., 2000; Naumann et al., 2002; Boskovic et al., 2014). However, and in contrast to the study reported by Boskovic et al. (2014) we observed a significant decrease in the number of BFCNs in both the SAMP8 and C57BL/6 mouse strains in aging mice. Quantification of the rate of the decreasing number of BFCNs reveals that they are lost at a faster rate, indicating an accelerated cell death of BFCNs in the SAMP8-p75^exonIII−/−^ mice. This decrease of cholinergic neurons correlates with an impairment in the Y-maze from 2 to 6 months.

To investigate what could be the mechanism of BFCNs loss in the long term, we rationalize that the loss of BFCNs might be due to the constitutive expression of a short isoform of p75^NTR^ previously described in the 129v background strain (von Schack et al., 2001). When analyzed by immunofluorescence using a specific antibody against the intracellular domain of p75^NTR^, we saw a significant labeling in the basal forebrain of the SAMP8-p75^exonIII−/−^ mice (Figure 5). Immunoprecipitation of total lysates from the basal forebrain supported the presence of a short-isoform of p75^NTR^, with a similar size as a p75-CTF construct used as a marker of migration (Figure 5). As p75-CTF is produced by the activation of TrkA signaling by NGF (Urra et al., 2007), we could consider the constitutive expression of p75-CTF in the SAMP8-p75^exonIII−/−^ as a gain-of-function of the TrkA/NGF and p75 signaling. It has been described that p75-CTF induces cell death of several neuronal types (Underwood et al., 2008; Skeldal et al., 2011; Vicario et al., 2015). Recently, we described that in the absence of a pro-survival signaling emanating from TrkA, p75-CTF induces the cell death of BFCNs in culture by activating p38, JNK, and caspase-3 pathway (Franco et al., 2021).

The SAMP8-p75^exonIII−/−^ mice could be a good model to study the consequences of cholinergic neurodegeneration in the context of pathological aging and high oxidative stress. Oxidative damage has been universally linked to AD (Guglielmotto et al., 2010; Cai et al., 2011) and is also observed in the SAMP8 mice (Morley, 2002; Pallas et al., 2008). p75^NTR^ has been involved in the cell death of sympathetic neurons upon oxidative stress (Kraemer et al., 2014a). Our results showed an increase in cell death in the SAMP8-p75^exonIII−/−^ mediated by the short isoform of p75^NTR^, supporting the cell death induced by p75^NTR^ signaling in conditions of high oxidative stress. This could explain why BFCNs degenerate faster or at a higher rate than the BFCNs from the C57/BL6 mice, with no oxidative stress.

Here we found an increase in the total expression of SREPB2 in the SAMP8-p75^exonIII−/−^ with respect to the SAMP8-p75^exonIII+/+^ and also in the C57BL/6- p75^exonIII−/−^ mice but at later time points (2 months versus 6 months) (Figure 6). This increase in the levels of SREBP2 parallels the increase of two of its main targets, the LDLR and HMGRC, suggesting an increase in the uptake and biosynthesis of cholesterol, respectively. Measurements of the total cholesterol levels in the basal forebrain suggested that the p75^exonIII−/−^ mice are prone to higher levels of free cholesterol in the basal forebrain. The brain is highly dependent on cholesterol (Petrov et al., 2016). The intact blood brain barrier (BBB) prevents the uptake of lipoproteins from the circulation in vertebrates. Unlike cholesterol in other organs in the periphery, brain cholesterol is primarily derived by *de novo* synthesis. During brain development, neurons have the capacity to synthesize their own cholesterol. In the adult state, however, cholesterol is synthetized by glia, mainly in the astrocytes, and transported bound with ApoE from the astrocytes to the neurons, that contain ApoE receptors like LDLR and LPR1 (Staurenghi et al., 2021; Li et al., 2022). The finding that the cholinergic neurons from the p75^exonIII^-KO mice re-express HGMRC suggests that the cholesterol homeostasis is disrupted somehow in these mice. An increase in the neuronal cholesterol content has been associated with some neurodegenerative diseases and cell death. In the Niemann-Pick disease Type-C (NPC), the impaired transport of cholesterol from the ER to the plasma membrane by defects in the Npc1 gene, induces an accumulation of intracellular cholesterol, endosomal alterations, and cell death (Cabeza et al., 2012). Previous reports described that excessive uptake, as well as synthesis of cholesterol, underlie neuronal cell death by a necroptosis-like mechanism (Funakoshi et al., 2016). These results suggest that the increased biosynthesis of cholesterol in the cholinergic neurons of the p75^exonIII−/−^ could be one of the mechanisms of BFCNs loss, although this hypothesis needs further research. Although we focus here on cholinergic neurons, we cannot discard that a similar phenotype is found in other neuronal populations that express p75 endogenously. However as cholinergic neurons express high levels of p75 during all the life the levels of the short isoform of p75 in the p75^exonIII^-KO might be higher in BFCNs than in cortical or hippocampal neurons that express much lower levels of p75 in the adult brain.

Previous data might suggest a cell-autonomous regulation of cholesterol synthesis genes by p75^NTR^. p75^NTR^ has been involved in the regulation of cholesterol synthesis in the forebrain (Yan et al., 2005; Korade et al., 2007) and in the liver (Baeza-Raja et al., 2016; Pham et al., 2019). Korade et al. (2007). described that the levels of p75^NTR^ positively correlate with the expression of cholesterol synthesis enzymes in both neuroblastoma cell lines and primary cerebellar neurons and ligand-activated p75^NTR^ mediates the activation of SREBP2 via p38 MAPK and caspase-2 in liver cell lines (Pham et al., 2019). Also, NGF, pro-NGF, and pro-BDNF induce the expression of LDLR in PC6.3 cells and in septal neurons in a TrkA and p75^NTR^-dependent manner (Do et al., 2016). Furthermore, in melanoma, metastasis is promoted by the upregulation of cholesterol synthesis genes by the axis NGF/TrkA/p75^NTR^ (Restivo et al., 2017). Our results suggested that the main role of TrkA is to facilitate the shedding of p75 to generate a p75-short isoform similar to p75-CTF, the real inducer of SREBP2 and HMGCR, as we showed by overexpression of p75-CTF in TrkA/p75-DKO PC12 cells. The upregulation of LDLR observed *in vivo* (Figure 6) was not observed in the PC12 cells system, suggesting that *in vivo* other more complex mechanism involving the regulation of cholesterol internalization is taking place.

All these data suggest that the constitutive expression of p75-CTF is behind the rapid cell death of BFCNs in the SAMP8 mice. A decrease in the number of BFCNs in the C57/BL6-p75^exonIII−/−^ mice background is also observed, and correlated with the presence of p75-CTF. Interestingly in the conditional p75^flox/flox^/ChAT-Cre mice, that do not express any domain of p75^NTR^, an absence of BFCN cell death during aging was not observed (Boskovic et al., 2014). One important read-out of these results is that the use of the p75^exonIII−/−^ should be cautious, as the expression of a pro-apoptotic short isoform may misinterpret the results and draw wrong conclusions regarding p75^NTR^ signaling, at least in the basal forebrain.

In summary, the generation of the SAMP8-p75^exonIII−/−^ mice described in this work uncovered a direct regulation of cholesterol synthesis genes by the TrkA/p75 axis and may facilitate the study of the degeneration of the cholinergic neurons by cholesterol dysregulation, a phenomenon also observed in several neurodegenerative diseases.

## Methods

### SAMP8 p75^exonIII −/−^ generation

SAMP8 mice were backcrossed with C57BL6 p75^NTR exonIII +/−^ mice (Lee et al., 1992) (Jackson Laboratories) for 12 generations to create a new SAMP8-p75 ^exonIII −/−^ strain. The animals were housed on a 12 h light/12 h dark circle with food and water provided *ad libitum* in specific pathogen-free (SPF) at constant 24 degrees temperature. All animal experimentation was controlled following the recommendations of the Federation of European Laboratory Animal Science Associations on health monitoring, European Community Law (2010/63/UE), and Spanish law (R.D. 53/2013) with approval of the Ethics Committee of the Spanish National Research Council (1,246/2022) and the local Government (2022-VSC-PEA-0139 type 2).

#### Brain fixation and tissue processing

Animals at the indicated age were perfused with PFA 4%, the brains were removed and postfixed from 2 h to overnight with PFA 4%, and next were washed several times with phosphate buffer (PB) 0.1 M pH 7.4 and cryoprotected overnight at 4°C with 30% sucrose in PB. After, the brains were frozen with Tissue-Tek compound OCT (Sakura) and cut into coronal sections at 10 μm with Leica CM1900 cryostat. Alternatively, brains were washed and sliced into 40-μm sections with Leica VT1200 vibratome and kept in PB 0.1 M with 0,005% sodium azide at 4°C until used.

#### Hippocampal astroglyosis

Inmunohistochemistry (IHC) of the astrocytic marker GFAP was carried out in the hippocampus. Cryostat sections were washed with PB 0.1 M and blocked with PB 0.1 M, 0.1%Triton X-100 and 3% Fetal Bovine Serum (FBS) for 60 min at room temperature. Sections were incubated 16 h at 4°C with rabbit antibody α-GFAP (DAKO, Z0334) 1:300 in blocking buffer. Next, slices were washed three times with PB 0.1 M and incubated with the secondary antibody Cy3 donkey α-rabbit (Jackson, 711–165-152) 1:500 for 2 hours. Nuclei were stained with nuclear marker 4,6-diamidino-2-phenylindoledihydrochloride (DAPI; Sigma) 1:1000 and washed again to finally cover them with a coverslip and Mowiol and DABCO (50 μL/mL). Images were captured with confocal microscope SP8 (Leica) and GFAP positive astrocytes of the CA1 area of the HC were analyzed by measuring the mean signal intensity per μm^3^. At least 5 slices per animal were measured. For this purpose, eight animals per condition were analyzed.

#### Hippocampal neurogenesis

For neurogenesis analysis in the dentate gyrus, four animals per group were used. 5-bromo-2′-deoxyuridine (BrdU) at 50 mg/kg was injected intraperitoneal three times every 2 hours and mice were sacrificed the following hour as described above. Sections corresponding to dentate gyrus (Bregma −1.3, −1.7, −2.1, −2.5), separated by 400 μm were subjected to BrdU along with Doublecortin (DCX) immunocytochemistry. DNA denaturalization was allowed by incubating the sections 20 min in HCl 2 N 37°C. Reaction was neutralized with borate buffer 0.1 M pH 8.5 for 10 min at room temperature. Tissues were blocked with PB 0.1 M, 0.2%Triton X-100, 10% FBS for 1 hour. Primary antibodies mouse α-BrdU (DAKO, M0744) 1:300 and goat α-DCX (Santa Cruz Biotecnhology, sc8066) 1:200 were added. Next day, slices were washed with PB 0.1 M and incubated with the secondary antibodies Alexa 488 donkey α-mouse (Invitrogen, A21202) 1:500 and Alexa 555 donkey α-goat (Invitrogen, A21432) 1:500. After 2 hours, nuclei staining and mounting were performed as described. Images of the dentate gyrus were captured with confocal microscope SP5 (Leica). Total dentate gyrus area was measured as dentate gyrus width multiplied by slice thickness and expressed in mm^2^.

#### Basal forebrain cholinergic counting

To count BFCNs, slices corresponding to basal forebrain and hippocampus were collected (Bregma 1.4 to −2.5 mm). 30 slices per animal, separated by 100 μm were observed at a fluorescence microscope (Leica), and the positive neurons were counted. To carry out the cholinergic neuron detection, Choline Acetyltransferase (ChAT) was used as cholinergic marker. The sections were blocked with blocking buffer PB 0.1 M, 1%Triton X-100, 3% FBS for 60 min at room temperature and incubated three overnights at 4°C with primary antibody goat antibody α-ChAT (Millipore, AB144P) 1:200. After 3 days, the antibodies were removed, the slides were washed three times with PB and incubated with biotin rabbit α-goat (Jackson, 305–065-046) 1:200 at room temperature for 1 h and posterior cy2 streptavidin (Jackson, 016–220-084) 1:200. Nuclei were stained and the slices mounted as described.

#### Inmunohistochemistry detection of p75, HMGCR and ChAT

Immunodetection was performed on vibratome slices. The chosen sections (around Bregma 0.86 mm) were treated with 10 mM Sodium Citrate pH 6.5 for 20 min at 85°C. Slices were then cooled down at room temperature and blocked with 0.1 M PB, 0.5% Triton X-100, 10% FBS for 1 h. After the blocking step, slices were incubated for 3 days in 120 μL of the primary antibody: mouse α-HMGCR (Abcam, 242,315) 1:100 or goat α-ChAT (Millipore, AB144P) 1:200 and rabbit α-p75 (Millipore, 07–476) 1:2000. After 3 days, the slices were washed 3 times with PB 0.1 M and incubated 2 h at room temperature with the secondary antibody. The following secondary antibodies were used, depending on the primary species: Alexa fluor 555 donkey α-mouse (Invitrogen, A31570) 1:500, Alexa fluor 488 donkey α-goat (Jackson, 705–546-147) 1:500, Alexa fluor 647 donkey α-rabbit (Invitrogen, A31573) 1:500 or alternatively Cy3 donkey α-rabbit (Jackson, 711–165-152) 1:500. The immunofluorescence reaction was carried out sequentially, first performing immunolabeling for HMGCR and repeating the incubation steps for ChAT and p75 individually. Nuclei staining and samples mounting were executed as previously described. Images were acquired with Confocal SP8 (Leica) with 40X magnification. To quantify the HMGCR signal in cholinergic neurons, the ChAT signal was utilized to generate masks. These masks were then transformed into regions of interest (ROIs) and applied to the HMGCR channel for signal quantification. The ImageJ software was employed for this analysis.

### Mice behavior tests

Groups of mice of 2 and 6 months conducted 3 different tests in the following order: open field, Y-maze test, and Novel Object Recognition test (NOR). Before performing the behavioral tests, the mice were moved to the behavioral room for habituation. In addition, each mouse was 5 min per day for 4 consecutive days with the experimenter. Every test was separated 3 to 5 days to let the mice rest.Y.

#### Open field test

Mice were placed individually into the periphery of a squared black box of 50×50 cm and 85 cm elevated from the floor for 5 min. They were free to explore, and they were recorded with an automatic activity monitoring system (Smart Video Tracking Software, PanLab). The area of the open field was divided into a 42×42 cm central zone (40% of the total surface) and a surrounded periphery zone. The following anxiety-related parameters were recorded: time spent and distance traveled in the center zone (and periphery). Total distance and mean velocity were used to assess general locomotion.

#### Spontaneous alternation Y-maze

Each arm of the Y-maze measured 32,5×8 cm. The mice were placed in the center and let explore freely for 8 min. Every time the mice put the four pawns on a new arm, it was counted as a new entrance. The correct alternations were counted for spatial memory parameters. The total number of entries was used to assess general locomotion.

#### Novel object memory test

Two days prior to the test, every mouse was placed in a 40×40 empty squared box for 10 min for habituation. 24 h later, training was conducted, the animals were placed in the same box containing 2 identical objects that they could explore for 10 min. The next day the test was conducted, in which one of the objects was changed for a novel one. The mice were recorded with a camera, and the time that the animal was exploring the novel and the familiar object was quantified.

### Cell culture

PC12 cells were cultured in DMEM (Gibco) supplemented with 10% FBS (Sigma), 1% Penicillin–Streptomycin, 1% L-Glutamine (Gibco) and 5% Horse Serum (Fisher, X) at 37°C in a humidified atmosphere with 5% CO_2_. Cells were treated with vehicle (DMSO), TrkA inhibitor, K-252a, (100 mM, Sigma, 05288), gamma-secretase inhibitor, Compound E, (10 μM, Millipore, 565,790), alpha-secretase inhibitor, TAPI (25 μM, Sigma, SML0739) and 3 h later NGF (100 ng/mL, alomone, N-245) or BDNF (100 ng/mL, alomone, B-250) was added. Cell lysis or fixation was performed 24 h later. For immunoblotting, cells were plated in p6-wells at 80% confluence and analyzed by western blot (as described above). For filipin staining, cells were plated in coverslips previously treated with Poly-d-lysine (10 mg/mL). In both assays, cells were allowed to attach and 24 h later and then starved with serum-free medium for 2 h hours.

### Filipin assay

A total of 2 × 10^4^ cells/well PC12 were seeded onto a sterile slide placed in a 24-well plate and incubated with the different inhibitor (see above). After 24 h the cells were fixed by 4% PFA for 1 h, the cells were stained for 2 h with filipin (50 μg/mL, Sigma) in 10% of PBS and propidum iodide was used for nuclear staining. UV filter was required to view the filipin staining (340-380 nm excitation, 40 nm dichroic, 430-nm long pass filter). The cells were protected from light during the procedure because the filipin fluorescence photobleaches very rapidly. A confocal microscope SP8 (Leica) was used to scan and record the fluorescence. For filipin intensity quantification, regions of interest (ROIs) were defined thresholding total projections of each condition and signal intensity was measured using ImageJ software.

### p75^NTR^ immunoprecipitation assay

Endogenous p75^NTR^-CTF was detected by immunoprecipitation. For that purpose, BF extracts were incubated with 500 μL TNE lysis buffer (50 mm Tris–HCl, pH 7.5, 150 mm NaCl, 1 mm EDTA, 0.1% SDS, 0.1% Triton X-100, 1 mm PMSF, 10 mm NaF, 1 mm Na_2_VO_3_, and protease inhibitor mixture) and disrupted by a dounce homogenizer. Samples were centrifuged and supernatant was subjected to immunoprecipitation. Extracts were incubated overnight with 1.5 μL of p75 antibody (Millipore, 07–476) at 4°C in an orbital shaker. The following day, 10 μL of previously TNE washed Protein G Agarose Beads (ABT, 4RRPG-5) were added and incubated for 2 h. Samples were then centrifuged to remove non-bounded proteins at 100 x g for 2 min to precipitate the agarose beads bound to the antibody, and washed three times in TNE lysis buffer with 0.2% Triton-X. Finally, for SDS-PAGE analysis, 30 μL reducing 2X sample buffer was added and the samples were boiled for 5 min at 96°C. Samples are centrifuged at 100 x g for 2 min and agarose beads are removed. Immunoprecipitated samples are then subjected to western blot analysis.

### Western blot

Mice were sacrificed with dislocation, and the BF was extracted. BF extracts were lysed for 30 min at 4°C in 200 μL of TNE. Protein concentrations were determined using the Bradford assay (Bio-Rad) and normalized across all samples. The lysate was subjected to centrifugation at 20,000 × *g* for 15 min at 4°C. The supernatant was removed, and 80–60 μg of protein were added to 4× SDS-sample buffer with 1.25% β-mercaptoethanol. Samples were denaturalized at 37°C for 15 min and resolved by SDS-PAGE. Proteins were transferred to nitrocellulose membranes, blocked with 5% BSA in TBS + 0.1% Tween (T-TBS) for 1 h and incubated overnight with the indicated antibodies. Primary antibodies used were the following: rabbit α-p75 (07–476, Millipore) 1:1000, mouse α- HMGCR (Abcam, ab242315) 1:1000, mouse α-LDLR (SantaCruz, sc-18823) 1:1000, mouse α-SREBP2 (SantaCruz, sc-13552) 1:1000, mouse α-actine (Sigma, A5441)1:5000. The membrane was incubated with the corresponding primary antibody diluted in T-TBS overnight at 4°C. For signal detection, secondary antibodies Alexa Fluor 800 donkey α-mouse (Invitrogen, A32789) 1:10000 and Alexa fluor 680 donkey α-rabbit (Invitrogen, A32802) 1:10000 were incubated for 1 h. After incubation with the appropriate secondary antibody, the membranes were imaged by LI-COR Odyssey scanner and quantified using ImageStudioLite (LI-COR).

### Statistical analysis

All the statistical analysis were performed with GraphPad Prism software. The results are represented as mean ± standard error of the mean (SEM). The normal distribution of all data sets were confirmed with the D’Agostino & Pearson test. To determine if the differences between 2 groups were significant the unpaired Student’s t-test was performed. For data presented as a fold increase, the One-sample *t* test was employed. For multiple comparisons one or two-way analysis of variance (ANOVA) test was used. Initially, it was evaluated if there were significant differences between the groups, then the Tukey’s post-hoc test was used to determine the specific differences between groups. In the plots the “*N*” indicates the number of the independent mice used of each strain and age for each experiment. In all the analysis a *p* value <0.05 has been considered statistically significant, and represented as: *p < 0.05; **p < 0.01; *** *p* < 0.001 and **** *p* < 0.0001.

## Data availability statement

The raw data supporting the conclusions of this article will be made available by the authors, without undue reservation.

## Ethics statement

The animal study was approved by Ethics Committee of the Spanish National Research Council (1246/2022) and the local Government (2022-VSC-PEA-0139 type 2). The study was conducted in accordance with the local legislation and institutional requirements.

## Author contributions

RC-B, JE-S, AB-M, MF, LC, and IC-B designed and performed experiments, acquisition of data. RC-B, HM, and MV edit the manuscript. HM and MV acquired funding. MV Conceptualization. All authors contributed to the article and approved the submitted version.
